# New standards for collecting and fitting steady state kinetic data

**DOI:** 10.3762/bjoc.15.2

**Published:** 2019-01-02

**Authors:** Kenneth A Johnson

**Affiliations:** 1 Department of Molecular Biosciences, The University of Texas, Austin, TX 78735, USA

**Keywords:** computer simulation, data fitting, enzyme catalysis, induced-fit, Michaelis constant, specificity constant

## Abstract

The Michaelis–Menten equation is usually expressed in terms of *k*_cat_ and *K*_m_ values: *v* = *k*_cat_[S]/(*K*_m_ + [S]). However, it is impossible to interpret *K*_m_ in the absence of additional information, while the ratio of *k*_cat_/*K*_m_ provides a measure of enzyme specificity and is proportional to enzyme efficiency and proficiency. Moreover, *k*_cat_/*K*_m_ provides a lower limit on the second order rate constant for substrate binding. For these reasons it is better to redefine the Michaelis–Menten equation in terms of *k*_cat_ and *k*_cat_/*K*_m_ values: *v* = *k*_SP_[S]/(1 + *k*_SP_[S]/*k*_cat_), where the specificity constant, *k*_SP_ = *k*_cat_/*K*_m_. In this short review, the rationale for this assertion is explained and it is shown that more accurate measurements of *k*_cat_/*K*_m_ can be derived directly using the modified form of the Michaelis–Menten equation rather than calculated from the ratio of *k*_cat_ and *K*_m_ values measured separately. Even greater accuracy is achieved with fitting the raw data directly by numerical integration of the rate equations rather than using analytically derived equations. The importance of fitting to derive *k*_cat_ and *k*_cat_/*K*_m_ is illustrated by considering the role of conformational changes in enzyme specificity where *k*_cat_ and *k*_cat_/*K*_m_ can reflect different steps in the pathway. This highlights the pitfalls in attempting to interpret *K*_m_, which is best understood as the ratio of *k*_cat_ divided by *k*_cat_/*K*_m_.

## Review

When Henri, Michaelis and Menten derived the equation for steady state enzyme turnover, they chose to define the rate in terms of *V*_max_ and the substrate dissociation constant for the hypothetical enzyme–substrate complex, *K*_S_ [1–2].


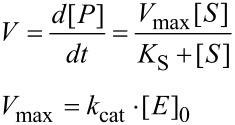


At the time, the choice of the terms *V*_max_ and *K*_S_ was logical because the concentrations of enzymes could not be determined and even the chemical makeup of enzymes was unknown. By including the unknown enzyme concentration in the term for the maximum velocity of turnover, the equation contained two variables, *V*_max_ and *K*_S_, consistent with the information content of the data and a minimal model.

In 1913, Michaelis and Menten provided evidence for the existence of an enzyme–substrate complex by careful rate measurements and rigorous quantitative analysis, fulfilling the major goal of their work [1–2]. Estimating the binding affinity for the substrate as *K*_S_ was an added bonus. These were profound discoveries that laid the foundation for enzymology throughout the 20th century.

The Michaelis–Menten equation was originally derived assuming that substrate binding was at equilibrium, and was later expanded by Briggs and Haldane [3] who used the steady state approximation to include the rates of substrate and product release in defining *K*_m_ according to a minimal model.


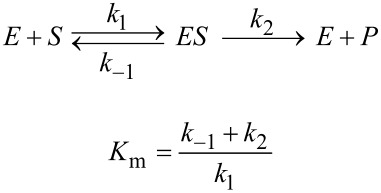


A century later, we know the structures of enzymes and can accurately determine their concentrations so we divide the measured rates by the known enzyme concentration to get the common form of the Michaelis–Menten equation:

[1] $$v=\frac{d[P]/dt}{{\left[E\right]}_{0}}=\frac{k_{\text{cat}}[S]}{K_{\text{m}}+[S]}$$

Using this equation, the two parameters derived in fitting data are *k*_cat_ and *K*_m_, from which we can calculate *k*_cat_/*K*_m_. However, *k*_cat_/*K*_m_ is the most important parameter as it is used to quantify enzyme specificity, efficiency and proficiency [4–5]. In fact, *k*_cat_ and *k*_cat_/*K*_m_ should be considered as the two primary steady state kinetic parameters, rather than *k*_cat_ and *K*_m_. A half century ago Cleland stressed that the two fundamental steady state kinetic parameters were *k*_cat_ and *k*_cat_/*K*_m_ and that *K*_m_ represents an “apparent dynamic dissociation constant under steady state conditions”, but noted that *K*_m_ is not an independent parameter [6]. This statement was based on the use of a Lineweaver–Burk (double-reciprocal) plot [7] to fit data where the intercept defines 1/*k*_cat_ and the slope defines 1/*k*_cat_/*K*_m_.


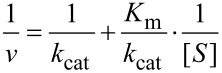


In Cleland’s analysis, the two primary steady state kinetic parameters were *k*_cat_ and *k*_cat_/*K*_m_ because they were the parameters derived in fitting data displayed on a double reciprocal plot. Today, the emphasis is on interpreting the steady state kinetic parameters in terms of enzyme structure and individual steps in the reaction pathway. This leads to a new justification for choosing *k*_cat_/*K*_m_ rather than *K*_m_ as a primary kinetic parameter. Of the three steady state parameters (*k*_cat_, *K*_m_, and *k*_cat_/*K*_m_) *k*_cat_/*K*_m_ is the most important as it quantifies enzyme specificity, efficiency and proficiency [4]. Moreover, both *k*_cat_ and *k*_cat_/*K*_m_ place lower limits on rate constants for individual steps in the pathway, while *K*_m_ is largely un-interpretable.

Cleland published the first computer programs [8] to fit data based on linear regression of data displayed on a double-reciprocal plot, and including a kind of global analysis in resolving steady state inhibition patterns, which are defined by the effects of inhibitors on the slope and intercept, i.e., *k*_cat_ and *k*_cat_/*K*_m_. However, there are serious disadvantages in using a double reciprocal plot due to the unequal weighting of errors and the compression of the most accurate data leading to a distorted view of the results. The unequal weighting of errors can be overcome if the standard deviations of the individual measurements are included in the linear regression analysis, but that is not always done.

**Figure 1:**
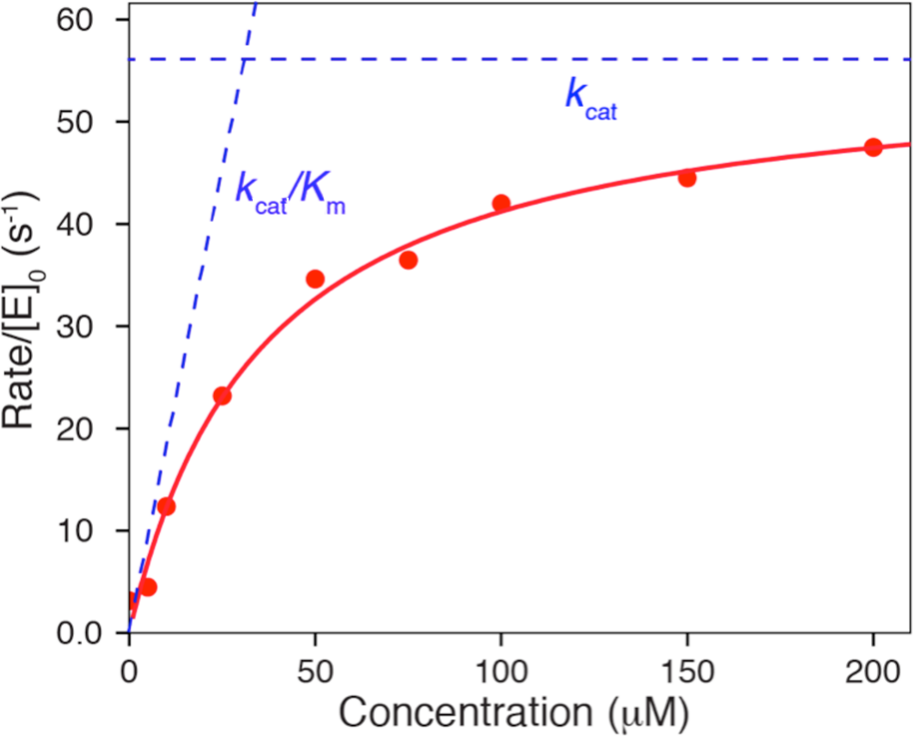
Michaelis–Menten plot. The rate of product formation is plotted versus substrate concentration and fit to a hyperbola. The dashed lines illustrate *k*_cat_/*K*_m_ (slope) and *k*_cat_. The intersection of the two lines gives the substrate concentration at which *k*_cat_/*K*_m_ [S]_i_ = *k*_cat_, so [S]_i_ = *k*_cat_/(*k*_cat_/*K*_m_) = *K*_m_.

Regardless of the method used to fit data, there is merit in fitting to derive *k*_cat_ and *k*_cat_/*K*_m_, rather than fitting to derive *k*_cat_ and *K*_m_ individually then calculating *k*_cat_/*K*_m_ from the ratio. There are large errors in *k*_cat_ and *K_m_* since these estimates each rely on extrapolation to infinite concentration of substrate, leading to larger errors in the calculated *k*_cat_/*K*_m_ value. On the other hand, the value of *k*_cat_/*K*_m_ is generally well defined from the initial slope of the concentration dependence, as illustrated in Figure 1. Thus, *k*_cat_/*K*_m_ can be understood as the apparent second order rate constant for substrate binding. More precisely, *k*_cat_/*K*_m_ is equal to the true second order rate constant for substrate binding to the enzyme multiplied by the probability that the bound substrate will be converted to product and released into solution. This principle can be illustrated using the simplest model:


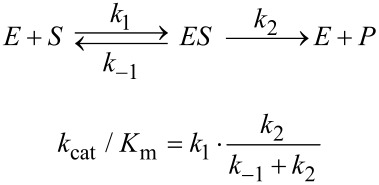


The term, *k*_2_/(*k_−_*_1_ + *k*_2_), gives the probability that the substrate reacts rather than dissociating. With more realistic models, the more complex equations for *k*_cat_/*K*_m_ can still be understood as the product of the rate constant for substrate binding times the probability of forward reaction.

Interpretation of steady state kinetic parameters takes on new significance in the current era of enzymology where the emphasis is on relating the parameters to individual rate constants and to structural and chemical transitions for each reaction in the pathway. While *k*_cat_/*K*_m_ can be directly interpreted in terms of enzyme specificity, it also provides a lower limit for the second order rate constant for substrate binding. Similarly, *k*_cat_ provides a lower limit for each first order rate constant following substrate binding through product release. On the other hand, the Michaelis constant cannot be interpreted unambiguously in the absence of additional information. In fact, *K*_m_ can be less than, greater than, or equal to the *K*_d_ for substrate binding. Here, the overly simplified model gives the wrong answer in suggesting that *K*_m_ is always greater than or equal to the dissociation constant (*K*_d_).


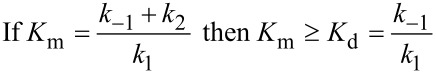


However, for a more complete model we come to a different conclusion:

[2] $$\begin{array}{c} E+S\;\overset{k_{1}}{\underset{k_{-1}}{⇄}}\;ES\;\overset{k_{2}}{\underset{k_{-2}}{⇄}}\;EP\;\overset{k_{3}}{\to }\;E+P\\ k_{\text{cat}}=\frac{k_{2}k_{3}}{k_{2}+k_{-2}+k_{3}}\\ K_{\text{m}}=\frac{k_{2}k_{3}+k_{-1}(k_{-2}+k_{3})}{k_{1}(k_{2}+k_{-2}+k_{3})}\\ k_{\text{cat}}/K_{\text{m}}=k_{1}\frac{k_{2}k_{3}}{k_{2}k_{3}+k_{-1}(k_{-2}+k_{3})} \end{array}$$

We can now see that depending on the intrinsic rate constants, *K*_m_ can be less than, greater than, or equal to the *K*_d_. Thus, in the absence of additional information, *K*_m_ cannot be interpreted to imply anything about the intrinsic rate and equilibrium constants governing enzyme catalysis. Although the *K*_m_ defines the concentration of substrate giving half maximal velocity, that is a phenomenological description without any mechanistic implications. On the other hand, *k*_cat_ and *k*_cat_/*K*_m_ provide meaningful lower limits on intrinsic rate constants.

The best understanding of *K*_m_ is as the ratio of *k*_cat_ and *k*_cat_/*K*_m_, so we consider that the Michaelis constant is a derivative of the two primary steady state kinetic parameters.


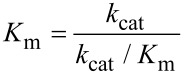


Although this statement appears as trivial algebra, it is profound because *k*_cat_ and *k*_cat_/*K*_m_ can reflect different steps in the enzyme pathway as will be described below. Moreover, it shows that the *K*_m_ value represents the balance point between the rate of turnover and the rate of substrate binding. The *K*_m_ represents substrate binding affinity only in the special case of rapid equilibrium binding.

A primary goal of fitting steady state data should be to accurately define *k*_cat_/*K*_m_. Rather than fitting to obtain estimates for *k*_cat_ and *K*_m_ and then calculating *k*_cat_/*K*_m_ as a ratio, a more accurate view is to consider *k*_cat_ and *k*_cat_/*K*_m_ as the primary steady state constants while *K*_m_ is obtained from their ratio. Traditionally, data have been fit using the standard form of the Michaelis–Menten equation to derive estimates of *k*_cat_ and *K*_m_ which are then used to calculate *k*_cat_/*K*_m_. However, there are often large errors in *k*_cat_ and *K*_m_ because these parameters are based on extrapolation to infinite substrate concentration, and these errors are compounded in calculating *k*_cat_/*K*_m_. Thus it is better to fit the data using an equation that provides *k*_cat_/*K*_m_ directly using the following form of the Michaelis–Menten equation:

[3] $$\begin{array}{l} v=\frac{k_{\text{SP}}[S]}{1+k_{\text{SP}}[S]/k_{\text{cat}}}\\ \text{where: }k_{\text{SP}}=k_{\text{cat}}/K_{\text{m}}\\ \text{or rather: }K_{\text{m}}=k_{\text{cat}}/k_{\text{SP}} \end{array}$$

We use the term *k*_SP_ = *k*_cat_/*K*_m_ to emphasize that the specificity constant (*k*_SP_) is a single parameter rather than a ratio and to stress that it represents the apparent second order rate constant for substrate binding. The use of the new term, *k*_SP_, overcomes the awkward use of *k*_cat_/*K*_m_, which is not only more difficult to say and write, but it also presents the mistaken impression that it is simply a function of the rate of enzyme turnover divided by the substrate binding affinity. The awkwardness is the result of historical precedent. Defining the specificity constant as *k*_cat_/*K*_m_ carries with it the baggage of thinking of the specificity constant as a ratio rather than a single parameter. Logic is influenced by the words we use to describe observations. It actually might help to avoid confusion in interpretation of *K*_m_ if we referred to the Michaelis constant as *k*_cat_/*k*_SP_.

### Measuring *k*_cat_/*K*_m_

**Figure 2:**
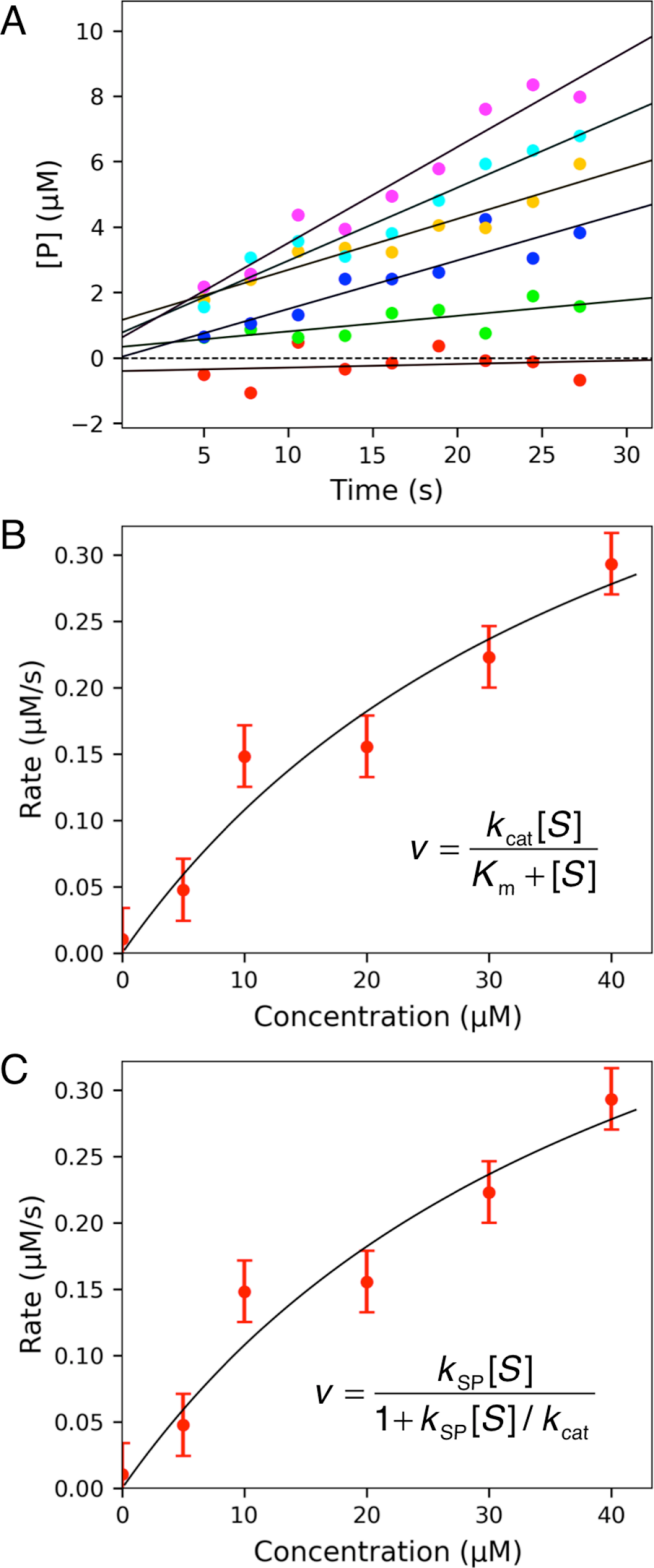
Fitting data to derive *k*_cat_ and *K*_m_. A) Synthetic data were fit to a straight line and then the observed rate was plotted versus substrate concentration (B, C). In B), the data were fit to standard Michaelis–Menten equation while in C), the data were fit to a modified form equation. The equations used are shown in each figure.

In order to get the best estimates of *k*_cat_/*K*_m_ from steady state kinetic data, it is preferable to fit the data to Equation 3 in which *k*_cat_ and *k*_cat_/*K*_m_ are the two fitted parameters rather than *k*_cat_ and *K*_m_ (Equation 1). To test this assertion, synthetic data were generated by computer simulation with *k*_cat_ = 50 s^−1^ and *K*_m_ = 20 μM. Data were generated at various concentrations of substrate (5, 10, 20, 30, and 40 μM), with a Gaussian distribution of random noise added to the data with a standard deviation of 0.5. The synthetic data were then fit to a straight line to estimate the rate (Figure 2A), which was then plotted versus substrate concentration and fit by nonlinear regression using either Equation 1 or Equation 3, defining *k*_cat_ and *K*_m_ or *k*_cat_ and *k*_cat_/*K*_m_*_,_* respectively.

As shown in Figure 2B,C, the fitted curves derived from either equation were indistinguishable, but as shown in Table 1 the error estimates in the fitted parameters were markedly different. The known standard deviation (sigma) values were included in the linear regression to estimate the rates and then the standard error estimates in fitted parameters were propagated to yield error estimates in *k*_cat_/*K*_m_. That is in computing *z* from the ratio of *x* and *y*, we compute the errors according to:


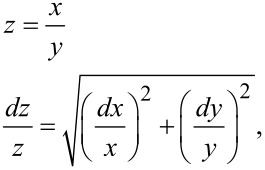


where *dx*, and *dy* represent the error estimates on the variables *x* and *y*, respectively.

**Table 1:** Summary of fitted parameters. Synthetic data were generated as described in the text and then fit to derive estimates of steady state kinetic parameters using different equations and different means of data fitting. Here we list the methods for fitting and the chosen fitted parameters for each method. The values for *k*_cat_, *K*_m_ and *k*_cat_/*K*_m_ are shown. Values in brackets were calculated from the other parameters. For example, in the first row, *k*_cat_/*K*_m_ was calculated from *k*_cat_ and *K*_m_, whereas in the second row, *K_m_* was calculated from estimates of *k*_cat_ and *k*_cat_/*K*_m_. Standard error estimates for calculated parameters were obtained by propagation of errors as described in the text. Note that the fitted parameters need not reproduce the input parameters used to generate synthetic because of the added errors and the limited set of data. A more complete data set with lower errors would return the input parameters.

method	fitted parameters	*k*_cat_ (s^−1^)	*K*_m_ (μM)	*k*_cat_/*K*_m_ (μM^−1^s^−1^)

equations	*k*_cat_ and *K*_m_	58.6 ± 25	44.4 ± 31	[1.32 ± 1.08]
	*k*_cat_ and *k*_SP_	58.7 ± 25	[44.4 ± 23]	1.32 ± 0.37

simulation	*k*_cat_ and *K*_m_	57.2 ± 5.9	27.2 ± 5.1	[2.11 ± 0.45]
	*k*_cat_ and *k*_SP_	57.3 ± 6.1	[27.8 ± 4.1]	1.62 ± 0.21

simulation full reaction	*k*_cat_ and *K*_m_	54.3 ± 4.9	23.6 ± 4.0	[2.30 ± 0.44]
	*k*_cat_ and *k*_SP_	55.2 ± 3.9	[24.9 ± 2.2]	2.22 ± 0.12

input values		50	20	2.5

Table 1 illustrates the improvements in error estimates when fitting the data to derive *k*_cat_/*K*_m_ (*k*_SP_) directly rather than calculating the value from the ratio of *k*_cat_ and *K*_m_. This is due to the large errors in estimating *k*_cat_ and *K*_m_ which are both based on extrapolation to infinite substrate concentration. In essence, the extrapolation errors are counted twice since they are reflected in both *k*_cat_ and *K*_m_ values. In contrast, when fitting to derive *k*_cat_ and *k*_cat_/*K*_m_, only *k*_cat_ is based on extrapolation while *k*_SP_ = *k*_cat_/*K*_m_ is obtained from the initial slope of the concentration dependence of the measured rate (Figure 1).

Admittedly, the “experiment” was set up to provide data only up to twice the *K*_m_ value to mimic those situations where the substrate concentrations available for testing are limited, so the exercise may not accurately reflect all laboratory settings. In that sense, the example may be biased in favor of fitting to derive *k*_cat_/*K*_m_ directly. However, as a counterpoint, the only “experimental errors” in the data are random since the added noise conforms to a normal distribution, so this may make the fitting to define *k*_cat_ and *K*_m_ more accurate than seen in the laboratory. The “experiment” was repeated three times by generating new synthetic data and then fitting the data to derive independent *k*_cat_ and *K*_m_ values. The averages from this analysis were *k*_cat_ = 45.4 ± 15.9 s^−1^ and *K*_m_ = 33.2 ± 18.3 μM, which give *k*_cat_/*K*_m_ = 1.36 ± 0.9 μM^−1^s^−1^. Averaging multiple experiments did not help to reduce errors as much as simply fitting data to a better equation.

One could argue that the choice of which equation to use is somewhat arbitrary. However, the common form of the equation was chosen over one hundred years ago for reasons that are no longer valid. Therefore, this historical precedent should no longer be followed given the advantage of fitting data to an equation that affords *k*_cat_/*K*_m_ directly.

### Fitting by simulation

Significant errors are introduced when fitting the primary data (product versus time) to a straight line because of the independent variables for slope and intercept for each trace. In fitting this data set with six concentrations of substrate and then fitting the rate versus concentration to a hyperbola, a total of 14 independent parameters were used. It is better to fit the data globally to derive only the two independent parameters from the primary data using computer simulation based on numerical integration of the rate equations [9].

In fitting steady state data by simulation, we start with the full realistic model for an enzyme-catalyzed reaction including five rate constants and then make approximations to simplify the model to be consistent with the information content of the data and the desired steady state parameters.


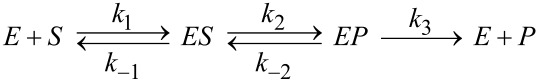


One could fit the data using all five rate constants, then calculate the steady state kinetic parameters from Equation 2. It is well known that steady state kinetics cannot define intrinsic rate constants; a corollary of that statement is that a large combination of intrinsic rate constants can be found to fit the data and provide estimates of steady state kinetic parameters. Thus, any combination of rate constants that fit the data provides valid estimates for the steady state parameters (*k*_cat_, *K*_m_ and *k*_cat_/*K*_m_). However, there will be large errors on each rate constant because of the large number of combinations of rate constants that can account for the data. The large errors would then propagate to large error estimates for each steady state parameter, which would not provide a realistic estimate of the uncertainty in measuring each parameter.

To limit the number of variables, we lock three of the parameters at reasonable values to reduce the model to only two unknowns. For example, we can modify the model to mimic rapid equilibrium binding. To do so, we use a conservative estimate for diffusion-limited substrate binding (*k*_1_ = 100 μM^−1^s^−1^) then make the chemistry step irreversible and product release at least 100-fold faster than *k*_2_ so that the rate of chemistry defines *k*_cat_. This represents the standard (often erroneous) interpretation of *k*_cat_ and *K*_m_. However, because we are only using this approximation to fit steady state kinetic data, this model need not to be true physically to give valid estimates of the steady state kinetic parameters. The simplified model shown below gives estimates of *k*_cat_ and *K*_m_ directly.

[4] $$\begin{array}{c} E+S\;\overset{100\;\mu {\text{M}}^{-1}{\text{s}}^{-1}}{\underset{k_{-1}}{⇄}}\;ES\;\overset{k_{2}}{\underset{0}{⇄}}\;EP\;\overset{>>k_{2}}{\to }\;E+P\\ k_{\text{cat}}=k_{2}\\ K_{\text{m}}=k_{-1}/100\;\mu {\text{M}}^{-1}{\text{s}}^{-1} \end{array}$$

We can also use an alternative form of the model to obtain estimates of *k*_cat_ and *k*_cat_/*K*_m_ directly. Here by setting *k_−_*_1_ = 0, after substrate binds it is always converted to product so *k*_cat_/*K*_m_ is defined by the value of *k*_1_. This model gives estimates of *k*_cat_ and *k*_cat_/*K*_m_ from the global fit.

[5] $$\begin{array}{c} E+S\;\overset{k_{1}}{\underset{0}{⇄}}\;ES\;\overset{k_{2}}{\underset{0}{⇄}}\;EP\;\overset{>>k_{2}}{\to }\;E+P\\ k_{\text{cat}}=k_{2}\\ k_{\text{cat}}/K_{\text{m}}=k_{1} \end{array}$$

Again, it is important to note that this need not represent physical reality in defining the intrinsic rate constants; the approximations are acceptable because we are only using the results to compute the steady state kinetic parameters. In fact, we illustrate below that either model can be used to fit the data to give identical steady state parameters although the standard errors will differ.

**Figure 3:**
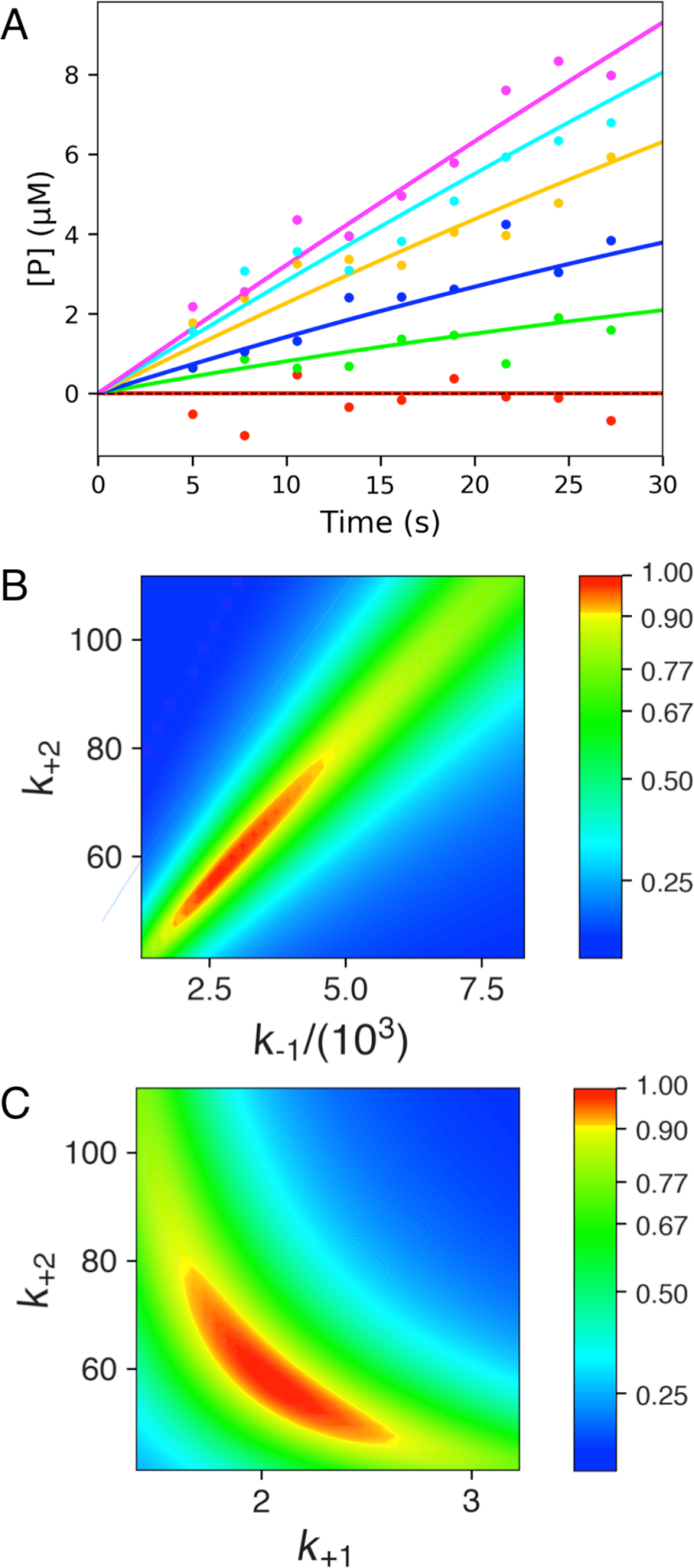
Fitting steady state data by simulation. A) Synthetic data from Figure 2A were fit globally to derive estimates of *k*_cat_ and *K*_m_ or *k*_cat_ and *k*_cat_/*K*_m_ as described in the text. B) Confidence contour analysis from fitting to derive *k*_cat_ and *K*_m_. C) Confidence contour analysis from fitting to derive *k*_cat_ and *k*_cat_/*K*_m_. The bar gives the color coding for the normalized χ^2^ values [10].

In Figure 3, we show the results of fitting the same data used in Figure 2. In Figure 3A, the curves represent the global data fit using only two unknown parameters, *k*_cat_ and *K*_m_ (Equation 4) or *k*_cat_ and *k*_cat_/*K*_m_ (Equation 5)*.* Because the results of the two fitting methods are indistinguishable graphically, we only show one figure to represent both methods (Figure 3A). However, as summarized in Table 1, the error estimates vary depending on the method used. As seen previously with equation-based data fitting, using the model to define *k*_cat_/*K*_m_ directly is more precise than computing *k*_cat_/*K*_m_ from individual estimates of *k*_cat_ and *K*_m_. It should also be noted that either method of fitting data by simulation is more accurate than the corresponding equation-based data fitting. This is because we are fitting the entire data set using only two parameters rather than fourteen. Using extraneous parameters introduces additional errors in data fitting.

Fitting by simulation also affords confidence contour analysis to more precisely estimate errors in data fitting and to reveal relationships between parameters [10]. In Figure 3B we show the confidence contour analysis from fitting to derive *k*_cat_ = *k*_2_ and *K*_m_ = *k*_−1_/*k*_1_. In this analysis, the two parameters are varied systematically and then we compute χ^2^, which we plot using a color scale to represent *z*-axis values on the plot of *k*_2_ versus *k*_−1_. The colors represent the values of χ^2^ normalized relative to the best fit so the red area defines the combinations of rate constants that give a good fit while the yellow band shows the χ^2^ boundary surrounding a good fit [10]. The elongated zone of good fit illustrates the linear relationship between *k*_2_ (defining *k*_cat_) and *k*_−1_ (defining *K*_m_). That is, this analysis clearly shows that the ratio of *k*_2_/*k*_−1_ is known with greater certainty than either parameter alone. In this simplified model *k*_cat_/*K*_m_ = *k*_2_*k*_1_/*k*_−1_ (*k*_1_ is fixed). Thus, the confidence contour analysis reveals that the data define *k*_cat_/*K*_m_ more accurately than either *k*_cat_ or *K*_m_.

The confidence contours for the global fit to derive *k*_1_ (*k*_cat_/*K*_m_) and *k*_2_ (*k*_cat_) are shown in Figure 3C. The curvature of the red area fits an equation of the form *k*_1_ × *k*_2_ = constant. This merely states that the net rate of product formation is a function of the combined rates of substrate binding and chemistry and that the net rate is known with greater certainty than either rate constant alone.

This analysis supports two important conclusions: (1) it is better to fit steady state data to define *k*_cat_ and *k*_cat_/*K*_m_ rather than *k*_cat_ and *K*_m_; and (2) simulation affords more accurate data fitting than the traditional methods of fitting to equations. Fitting data to equations necessarily involves limitations to conform to the approximations in defining the initial velocity of turnover before the substrate is consumed and product builds up, and it requires that the data be fit a second time in the form a plot of estimated rate versus concentration. Fitting by computer simulation overcomes these limitations.

### Full progress curve analysis

**Figure 4:**
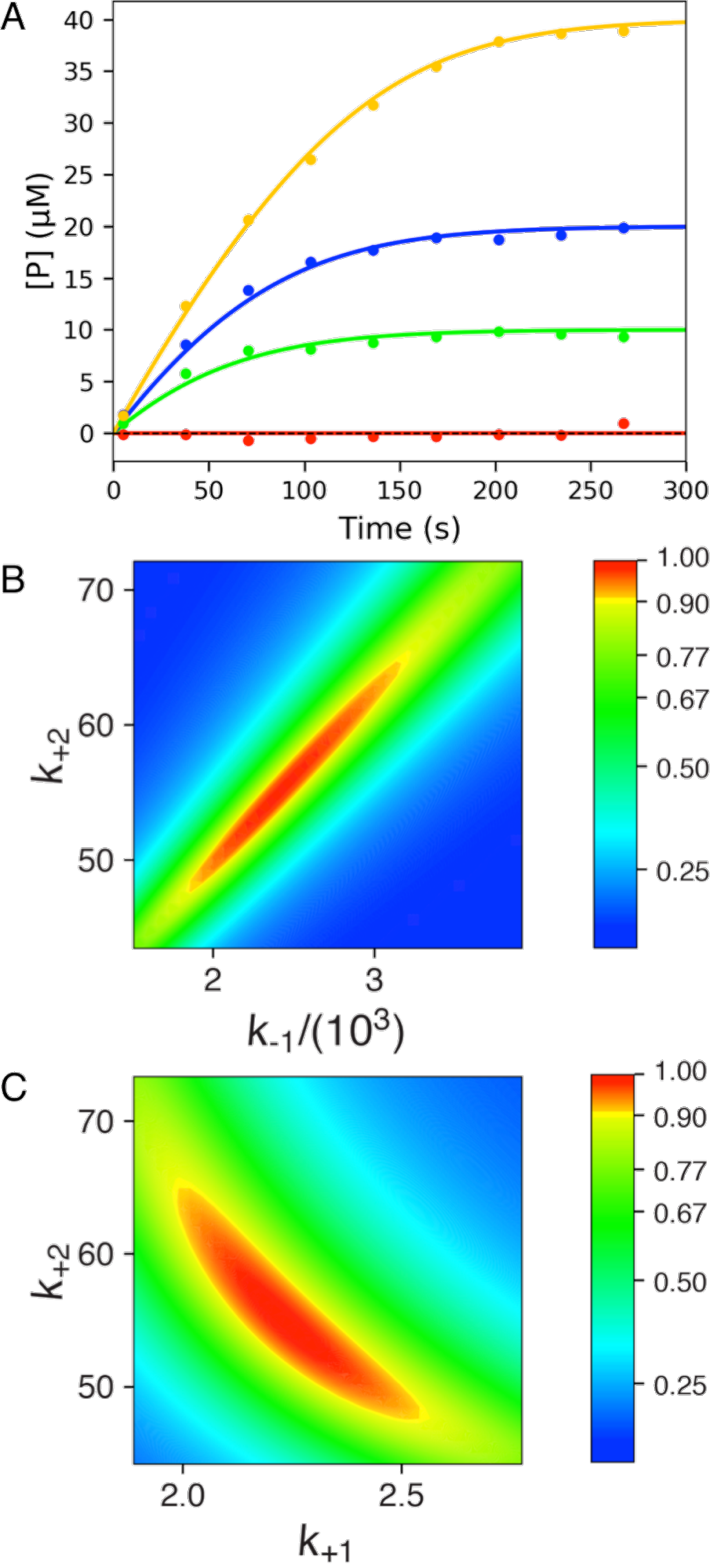
Fitting full progress curve data by simulation. A) Synthetic data were generated and then were fit globally to derive estimates of *k*_cat_ and *K*_m_ or *k*_cat_ and *k*_cat_/*K*_m_ as described in the text. B) Confidence contour analysis from fitting to derive *k*_cat_ and *K*_m_. C) Confidence contour analysis from fitting to derive *k*_cat_ and *k*_cat_/*K*_m_. The bar gives the color coding for the normalized χ^2^ values [10].

The ability to fit data by simulation based on the numerical integration of rate equations frees the experimentalist from the confines of initial velocity measurements. One can easily follow the reaction to completion beyond the linear phase and even fit the entire time course to derive estimates of *k*_cat_ and *k*_cat_/*K*_m_. To illustrate this, we simulated ten data points at the same concentrations of substrate examined in Figure 2, but here we allow the reaction to go to completion (Figure 4A). The same standard deviation (0.5) now leads to less apparent noise because of the larger signal. The synthetic data were then globally fit to derive estimates of either *k*_cat_ and *K*_m_ (Equation 4) or *k*_cat_ and *k*_cat_/*K*_m_ (Equation 5). Like before, the choice of method for data fitting did not affect the appearance of the fitted curves so we show only one (Figure 4A). However, the confidence contour analysis again shows the linear relationship between *k*_2_ (defining *k*_cat_) and *k*_−1_ (defining *K*_m_), demonstrating that *k*_cat_*/K*_m_ is more accurately defined by the data than either constant individually (Figure 4B). This analysis also revealed the lower standard errors estimated for *k*_cat_/*K*_m_ measured directly compared to values calculated from the ratio of *k*_cat_ and *K*_m_ (see Table 1).

Analysis of full progress curve kinetics provides the most accurate estimates. Given the experimental constraints of limited substrate concentrations and the same number of data points collected, it is better to spread the data points out and follow the reaction to completion rather than restrict the measurement to the initial velocity. One could stop data collection at any time and still be able to fit the data without regard for maintaining initial velocity conditions. Fitting data as was done by Michaelis and Menten more than 100 years ago imposes significant limitations on the quality of data that can be collected because it restricts data fitting to the earliest part of the curve with the only small amounts of product formed. It is more accurate to monitor the reaction for longer times, allow the reaction rate to fall off as substrate is consumed but account for the deviation from linearity by fitting the data using computer simulation. Product inhibition can also be resolved if it contributes significantly to the data [11].

### Standards for data collection

When it is possible, data should be collected at substrate concentrations exceeding the *K*_m_ by at least 4-fold so that the data reach 80% saturation. Concentrations 9-fold greater than the *K*_m_ are required to reach 90% saturation. The question of how high to go in substrate concentration must also be considered relative to the availability and solubility of the substrate. The standard rules apply for measurement of initial velocities requiring that less than 10% of the substrate should be consumed during the measurement to support the steady state approximation. Of course, this requirement does not apply if the data are fit by computer simulation, so more accurate data can be obtained based on formation of a larger signal in measuring product formation.

It is always important to carefully select the minimal number of measurements to provide the needed information to optimally use limited resources. Here, full time course measurements are by far the best, as described above. In the absence of product inhibition, steady state kinetic parameters can be derived from a single sample starting at a substrate concentration 8–10 fold higher than the *K*_m_ and following the reaction to completion. To test for product inhibition, two replicates at lower substrate concentrations will suffice. At larger substrate concentrations, larger concentrations of product formed toward the end of the reaction alter the rate of approach to equilibrium if the rebinding of product to the enzyme occurs appreciably. Globally fitting measurements at three substrate concentrations may be sufficient to define *k*_cat_ and *k*_cat_/*K*_m_ and *K*_i_ for product inhibition. The ready availability of computer programs for fitting by numerical integration of the rate equations renders the initial velocity measurements obsolete.

**Figure 5:**
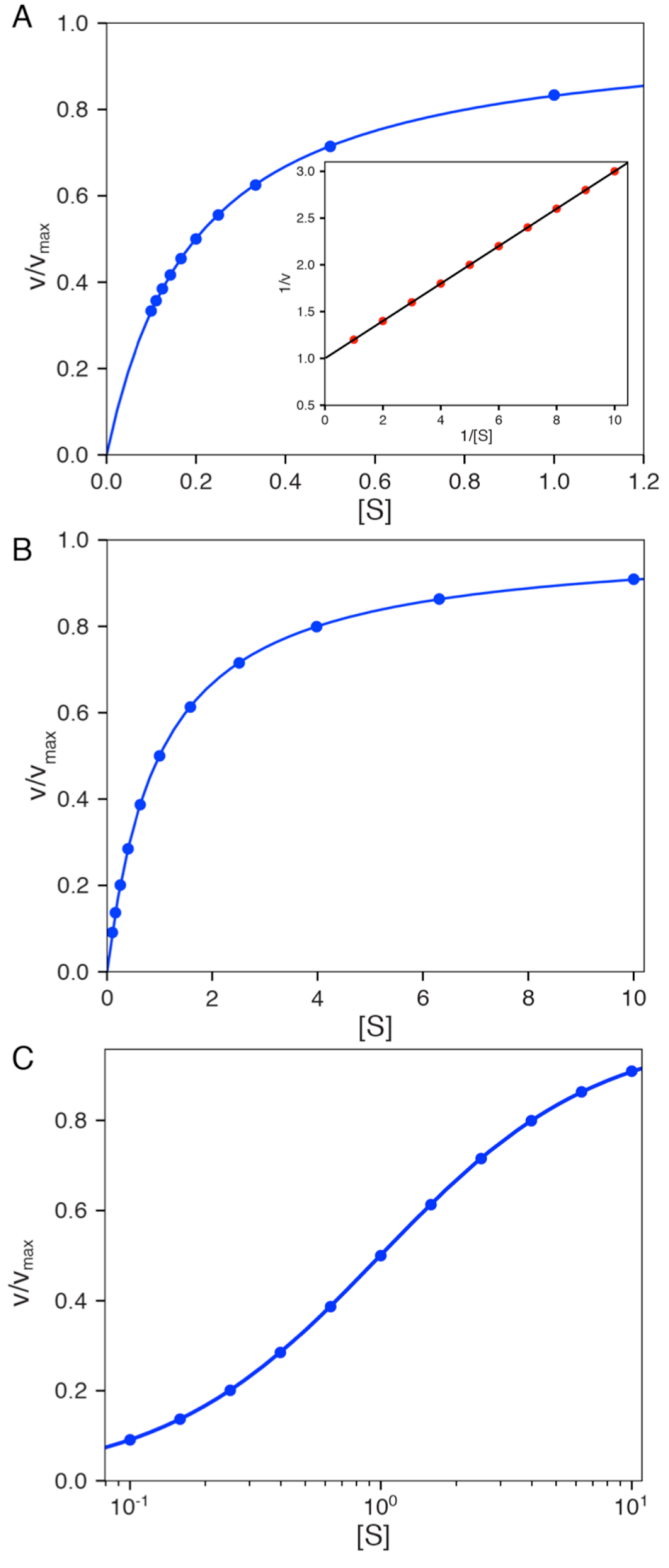
Optimal spacing of data points. Different scenarios for computing the distribution of data points for steady state measurements are shown. A) Linear distribution on a double reciprocal plot. Sample data were calculated with *k*_cat_ = 1 s^−1^ and *K*_m_ = 0.2 (arbitrary units). The inset shows the same data on a double reciprocal plot. B) Logarithmic distribution of data points. Sample data were calculated with *k*_cat_ = 1 s^−1^ and *K*_m_ = 1 (arbitrary units). C) Data in B on a logarithmic *x*-axis. The smooth lines show the fitted curve.

In setting up initial velocity measurements one must first decide on the range of concentrations to use and whether to space the points evenly. It is generally accepted that concentrations should be more closely spaced below and slightly above the *K*_m_ and spaced further apart at higher concentrations. The data at low concentration define *k*_cat_/*K*_m_ while the data at the higher concentrations are only needed to extrapolate to get *k*_cat_. Cleland has suggested collecting data with points evenly spaced on a Lineweaver–Burk plot [12]. However, this conclusion represents a mistake rooted in the distortion of the data when viewed on a double reciprocal plot as shown in Figure 5A. Spacing points evenly on a double reciprocal plot does not provide the best distribution of data given the importance of accurately defining *k*_cat_ and *k*_cat_/*K*_m_. A better alternative is to space points evenly on a logarithmic scale (Figure 5B,C). Here 11 points can be distributed on a log scale with [S]/*K*_m_ ratios of 0.16, 0.25, 0.4, 0.6, 1, 1.6, 2.5, 4, 6 and 10 (rounded off). This provides a convenient series that most accurately defines both *k*_cat_ and *k*_cat_/*K*_m_. These guidelines are predicated on having an estimate of *k*_cat_ and *k*_cat_/*K*_m_ before setting up the measurements. Since all experiments must be replicated prior to publication, the first experiment can be used to explore the range of concentrations and time for data collection. A second experiment can then be designed based upon the initial estimates to achieve an optimal distribution of data points to get publication quality data.

Another question is whether to collect triplicates at each concentration or to sample at three times as many concentrations. Because all independent measurements are treated equally in fitting by nonlinear regression, it is better to collect data at more concentrations rather than in triplicate at fewer concentrations. The average standard deviation of the measurements is evident in the scatter of the data from the fitted curve and can be estimated from the χ^2^ valued derived in fitting the data.

### Active site concentration

Interpretation of steady state turnover rates is dependent on an accurate estimate of the concentration of active sites. There are significant systematic errors in measurements of protein concentrations using dye-binding assays or by absorbance measurements, and the fraction of protein that is active is not known without direct measurement. For these reasons, it is important to perform an active site titration to establish the concentration of active sites. One method is isothermal titration calorimetry relying on the heat change upon binding of a substrate analog. Because the method is relatively insensitive it requires high concentrations of protein (usually μM) so the stoichiometry is easy to determine when titrating with a known concentration of a substrate analog [13]. In addition, many proteins show a change in fluorescence (tyrosine and tryptophan residues) upon substrate binding, affording accurate measurements of the stoichiometry and dissociation constant for binding from an equilibrium titration [14]. Other methods, such as rapid gel filtration and filter binding assays are limited by the rate of ligand dissociation relative to the time required to perform the separation. Alternatively, the kinetics of a pre-steady state burst of product formation can be used to estimate the concentration of active sites under favorable conditions [15]. In any event, kinetic data should be normalized by dividing the rate by the concentration of enzyme active sites, and the basis for estimating enzyme concentration should be clearly stated. It is no longer acceptable to report enzyme specific activity in units of product/min/mg of enzyme; rather, report values of *k*_cat_ and *k*_cat_/*K*_m_.

### Interpretation of *k*_cat_/*K*_m_

The steady state kinetic parameter, *k*_cat_/*K*_m_ is not merely the ratio of *k*_cat_ and *K*_m_; rather, it should be considered as a single parameter because it quantifies enzyme specificity, efficiency and proficiency [4–5]. Intuitively, it may seem that the substrate with the greater *k*_cat_ reacts faster and would be preferred, but that is not necessarily the case when two competing substrates are present as the one with a lower *K*_m_ would occupy more of the enzyme. However, simple algebra shows that the relative rate of turnover of two competing substrates is defined by their relative concentrations and *k*_cat_/*K*_m_ values for substrates A and B.


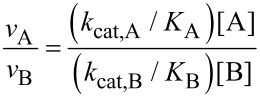


Thus, enzyme specificity is quantified by *k*_cat_/*K*_m_. It is for this reason that *k*_cat_/*K*_m_ is called the specificity constant. Specificity is a function of the apparent second order rate constants for substrate binding and conversion to product. When competing substrates are both present, the one that binds to the enzyme the fastest and is then converted to product wins the competition. In contrast, *k*_cat_ defines how fast the enzyme catalyzes a given reaction, not which substrate the enzyme prefers.

Although *k*_cat_/*K*_m_ provides a quantitative measure of enzyme specificity, it does not define the underlying basis for specificity. Therefore, a major effort is currently underway to understand how elementary steps in catalysis contribute to the observed specificity and to understand how enzymes evolve to acquire new specificities. Analysis of enzyme families has revealed that members within a family share a common catalytic core and a variable loop domain that closes over the substrate and confers substrate specificity [16–17]. Moreover, specificity could be dependent on conformational changes in the loop domain after substrate binding. The concept of induced-fit, where the substrate induces a change in enzyme structure to align catalytic residues, was first proposed in somewhat vague terms by Koshland [18] in an attempt to understand how an enzyme can exclude a smaller substrate than the preferred one.

The induced-fit model proposes a two-step binding pathway in which the substrate first binds to an open form of the enzyme and then the enzyme closes leading to tighter binding and organization of catalytic residues.


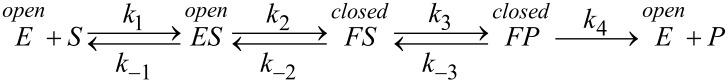


For decades debate raged as to whether a two-step binding mechanism could lead to increased enzyme specificity. Most notably, Fersht argued that because a two-step binding sequence has the same net free energy change as a corresponding one-step mechanism, a two-step binding sequence could not lead to greater enzyme specificity [5]. This logic is flawed because it follows from the simple definition of *K*_m_ as equal to the *K*_d_ for substrate binding and assumes the conformational change step is fast and at equilibrium preceding catalysis. Thus, the conclusion is a restatement of the assumptions used to define the model.

More recently, Warshel has asserted that pre-chemistry barriers cannot contribute to enzyme specificity unless they are rate limiting [19]. In his arguments, Warshel fails to appreciate the distinction between steps in the pathway that govern specificity (*k*_cat_/*K*_m_) versus those that govern the net turnover rate (*k*_cat_). The terminology in which the specificity constant is given by the ratio of *k*_cat_ divided by *K*_m_ contributed to the confusion. Throughout his recent paper, Warshel continually referred to the rate-limiting step as if it also defined specificity. In general, it does not.

To resolve this controversy, a direct measurement of the rates of the conformational change and the chemical reaction at the active site of the enzyme was required. Steady state kinetic methods do not suffice. Transient state kinetic analysis are needed to measure events occurring during a single enzyme cycle, but in the end, we must account for steady state kinetic parameters calculated from intrinsic rate constants. Resolution of the longstanding controversy over the role of induced-fit in enzyme specificity illustrates the importance of properly interpreting *k*_cat_ and *k*_cat_/*K*_m_ based on asking how each step in the reaction contributes to the observed *k*_cat_/*K*_m_ values for the correct and incorrect substrates.

**Figure 6:**
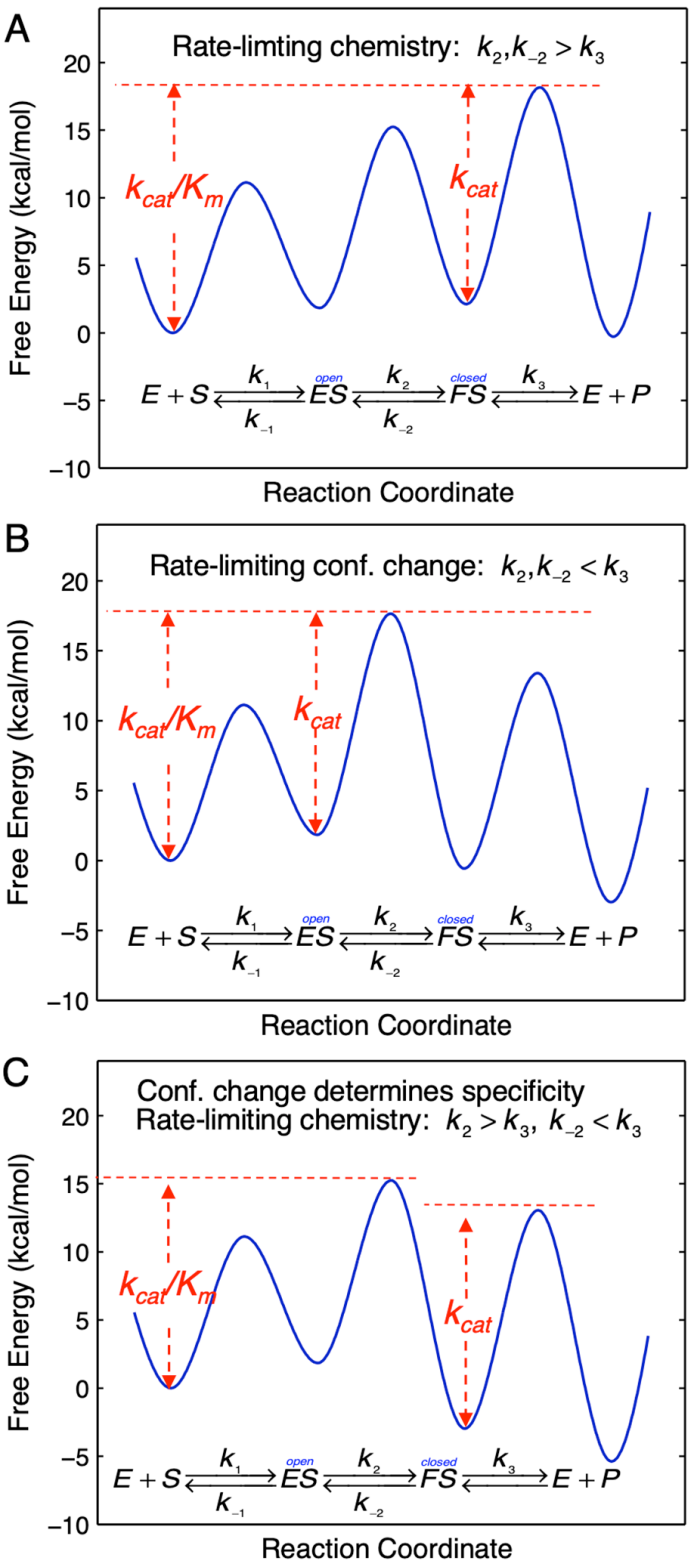
Free energy profiles. Free energy profiles are shown for a three step model with different rate constant relationships as described in the text and summarized on each figure. The free energy profile was calculated using transition state theory: Δ*G*^‡^ = −*RT*·ln(*k*/(*k*_B_*T*/*h*)), where *k* is the rate constant, *k*_B_ is the Boltzmann constant and *h* is Planck’s constant. Second order rate constants were converted to pseudo-first order rate constants using an estimated physiological concentration of substrate.

Figure 6 shows three possible scenarios for the effect of the conformational change on *k*_cat_ and *k*_cat_/*K*_m_. In this figure, we show free energy profiles computed from different combinations of rate constants for a minimal three-step reaction where product release is fast after the chemistry step.


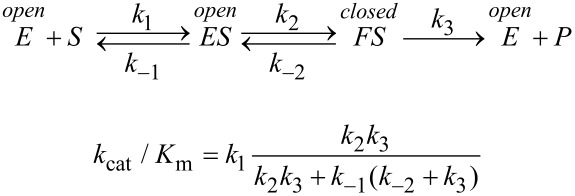


In each figure, the slow step in the pathway defines *k*_cat_ and is identified as the step with the largest local barrier (relative to the local minimum) in the free energy profile. On the other hand, the specificity constant, *k*_cat_/*K*_m_, can be identified as the steps leading from the starting state up to the highest overall barrier.

#### Case 1

In Figure 6A, *k*_cat_/*K*_m_ and *k*_cat_ are both governed by the chemistry step. In this model, the initial binding and conformational change are both rapid equilibrium reactions leading up to chemistry. In this case, *k*_cat_ and *k*_cat_/*K*_m_ can be approximated as follows (Note *K*_i_ = *k*_i_/*k*_−i_):


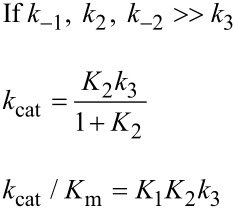


Note that *k*_cat_ is not simply defined by *k_3_*; rather, the equilibrium constant for the conformational change step defines the fraction of the bound substrate that is in the *FS* state (*K*_2_/(1 + *K*_2_)). An unfavorable equilibrium constant for the conformational change (*K*_2_) could reduce both *k*_cat_ and *k*_cat_/*K*_m_.

#### Case 2

We next consider the case shown in Figure 6B where the conformational change is rate-limiting. Here it can be seen that the rate of the conformational change governs both *k*_cat_ and *k*_cat_/*K*_m_.


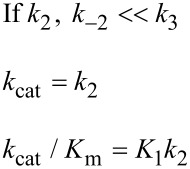


This model mimics the standard view of catalysis with a single equilibrium binding step followed by a single rate limiting step, but in this case, the conformational change, not chemistry, is rate limiting.

#### Case 3

Finally, we consider the case where chemistry is rate-limiting, but the reverse of the conformational change step is slower than the rate of chemistry (Figure 6C). Here we see that the conformational change step governs specificity (*k*_cat_/*K*_m_) but the rate of chemistry governs *k*_cat_.


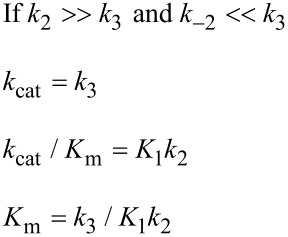


This leads to a surprising result that had not been anticipated in decades of research. To fully understand this, it is instructive to examine the equation for *k*_cat_/*K*_m_ calculated from the three-step model (Equation 2).


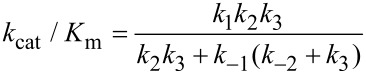


When *k*_−2_ << *k*_3_, this reduces to:


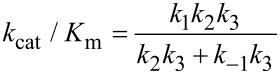


By dividing the numerator and denominator by *k*_3_, this reduces to an equation that no longer includes *k*_cat_ (*k*_3_).


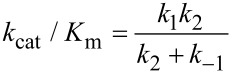


This equation can be further reduced by assuming that the substrate binding to the open state is in rapid equilibrium, i.e., *k*_−1_ >> *k*_2_.


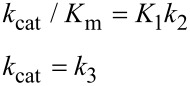


This leads to the surprising result that the *K_m_* is defined by:


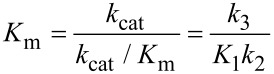


The product, *K*_1_*k*_2_ defines the second order rate constant for substrate binding. Thus the *K*_m_ is defined the balance between the rate of enzyme turnover relative to the rate of substrate binding. Because the reverse of the conformational change step is very slow, the two-step binding reaction does not come to equilibrium. Rather, the substrate binds and the enzyme closes leading to rapid catalysis and product release. Because the reverse of the conformational change step is so much slower than chemistry, the initial weak substrate binding and the conformational change are the primary determinants of specificity.

### DNA polymerase fidelity

DNA polymerases provide ideal model systems to study enzyme specificity because fidelity is high and physiologically relevant, and the alternate substrates are well known. Moreover, it is easy to perform single turnover kinetic measurements to examine steps leading up to the chemical reaction by mixing an enzyme DNA complex with only one nucleoside triphosphate. Recent work on DNA polymerase fidelity has shown that the rate of the conformational change from open to closed state is much faster than chemistry [20–21]. If we were only concerned with defining the rate-limiting step (*k*_cat_) we would stop at this point and simply conclude that chemistry was rate limiting; and since *k*_cat_/*K*_m_ defines specificity, the chemistry step must also define specificity. However, that would be wrong. An additional experiment was required to measure the rate of substrate release using dideoxy-terminated DNA to allow the conformational change but prevent chemistry. This experiment allowed the measurement of the rate of enzyme reopening to release substrate before chemistry. The results showed that once the enzyme closes over a correct substrate, it almost always continues to react rather than release the bound substrate. Globally fitting multiple experiments yielded the following rate constants [21]:


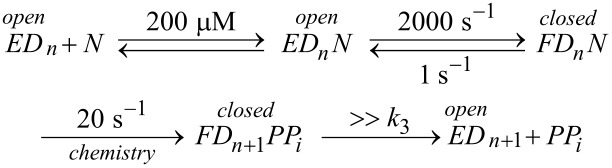


where *ED_n_* represents an enzyme–DNA complex in the *open* state with a primer strand *n* residues long, while *FD_n_N* represents the closed state with nucleotide (*N*) bound. Note that we show 1/*K*_1_ = 200 μM for the initial weak binding step.

The initial weak binding of nucleotide to the open state (*K*_d_ = 200 μM) is followed by a very fast conformational change to the closed state to afford a net *K*_d_ = 1/(*K*_1_(1 + *K*_2_)) = 200 nM. Because the chemistry step (*k*_3_) is so much faster than the rate at which the enzyme opens to release the substrate (*k*_−2_), the *FD_n_N* state goes forward 95% of the time. Thus, the conformational change is the primary determinant of enzyme specificity because it commits the substrate to forward reaction. For the DNA polymerase studied, the rate of product release is much faster than chemistry so the model reduces to a three-step model. Accordingly the specificity constant is defined by the two-step binding reaction, while *k*_cat_ is defined by the rate of the chemical reaction.

**Figure 7:**
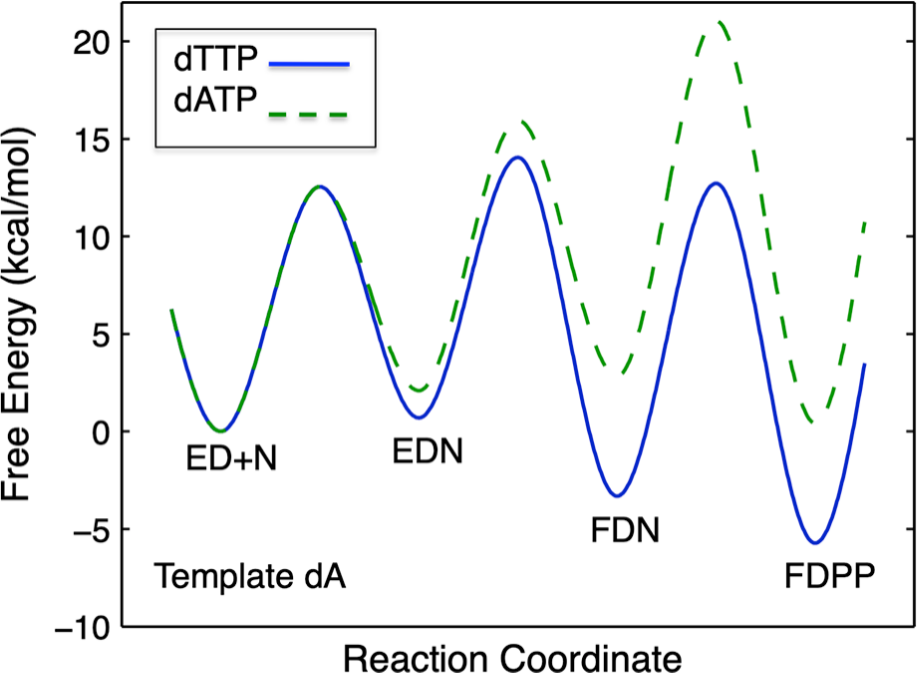
Free energy profile for DNA polymerization. Free energy profiles for a correct base pair (solid blue line) and a mismatch (dashed green line) were computed from data presented in [21].

This result was a big surprise, which had not been anticipated in attempts to foresee the ways in which induced-fit could contribute to specificity [22]. For 20 years numerous investigators in the DNA polymerase field had attempted to resolve whether the conformational change or chemistry was rate limiting. We had neglected to measure the rate of the reverse of the conformational change (enzyme opening to allow release of bound substrate) relative to the rate of chemistry, and that proved to be the key to understanding specificity. As shown in Figure 7, the free energy profile shows that after the conformational change, the enzyme is committed to go forward because there is a larger barrier to going backwards. The highest overall barrier is the conformational change step, thus defining the specificity constant [21,23–24].


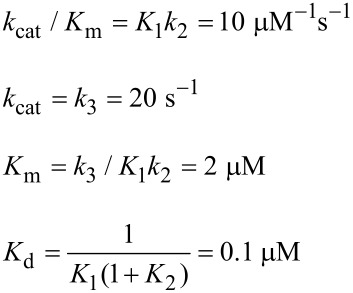


We compare a free energy profile for correct nucleotide incorporation with that for a mismatch (Figure 7). With a mismatch (dashed line), the chemistry step becomes very slow, while the rate of enzyme opening is much faster. Thus, for a mismatch, the conformational change step comes to equilibrium prior to rate-limiting chemistry. In this case, the chemistry step governs both specificity and rate-limiting steps.


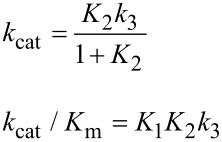


Mismatch recognition by the enzyme leads to a change in the specificity-determining step, but not the rate-limiting step.

We can now understand that the conformational change is the major specificity-determining step. The substrate binds weakly and then the enzyme closes. If the substrate shows the right geometry (structurally and electrostatically) the closed state is stabilized and organization of catalytic residues leads to fast catalysis. If the substrate is not the right size and shape, the enzyme fails to close tightly, chemistry is slow, and the enzyme rapidly opens to release the mismatched substrate [23–24].

This new paradigm for enzyme specificity provides a very satisfying resolution of the long-standing controversy over the role of induced fit in enzyme specificity. The conformation change serves as a gate-keeper to facilitate catalysis of the favored substrate while promoting release of alternate substrates.

## Conclusion

This short review shows that the traditional Michaelis–Menten equation defined in terms of *k*_cat_ and *K*_m_ should be replaced by one in which the two variable parameters are *k*_cat_ and *k*_cat_/*K*_m_. There are two reasons for this change: (1) *k*_cat_/*K*_m_ is the most important steady state kinetic parameter because it quantifies enzyme specificity, efficiency and proficiency; and (2) there are smaller errors in fitting to derive *k*_cat_/*K*_m_ directly rather than by calculation of the ratio of *k*_cat_ and *K*_m_ derived independently in fitting steady state kinetic data. In addition, there are significant advantages in fitting by computer simulation rather than in using the conventional approach using equations. Instead of fitting steady state data to a straight and then fitting the concentration dependence of the observed rate, the raw data can be fit directly in a single step with fewer unknown variables, resulting in less error on the estimates for steady state kinetic parameters.

The use of the ratio *k*_cat_/*K*_m_ to describe the specificity constant has long been a source of confusion. We now recognized that *k*_cat_/*K*_m_ and *k*_cat_ can reflect different steps in the enzyme pathway. Although *k*_cat_ is a function of rate limiting steps in the pathway, steps defining *k*_cat_/*K*_m_ establish specificity and need not be identical to the rate-limiting steps. Here the longstanding use of *k*_cat_/*K*_m_ as the specificity constant gets in the way of proper understanding because, of course, one expects that *k*_cat_ is part of *k*_cat_/*K*_m_ so they must be measuring the same step. This simplified logic overlooks the situation where *k*_cat_ in both the numerator and denominator of *k*_cat_/*K*_m_ cancel so that the ratio is no longer related to *k*_cat_; such is the case for DNA polymerase specificity.

Results presented here also document the advantages of fitting kinetic data using computer simulation based on the numerical integration of rate equations. Beyond what is shown here, one can also simultaneously fit steady state data along with equilibrium binding and pre-steady data kinetic data to derive a single unifying model to account for all of the results. This approach provides the most robust and accurate method for data fitting to ensure that the model fully accounts for all experimental observations. Moreover, confidence contour analysis provides a critical check to show the extent to which the fitted parameters are constrained by the data, and thereby support the model.

## Financial Conflict of Interest Statement

KAJ is President of *KinTek Corporation* which licenses the *KinTek Explorer* software described in this review.
